# What are the home care needs of Chinese healthcare workers: a latent class analysis

**DOI:** 10.3389/fpubh.2024.1369456

**Published:** 2024-08-19

**Authors:** Luling Zhou, Suzhen Liu, Hang Li

**Affiliations:** ^1^West China Hospital, Sichuan University/West China School of Nursing, Sichuan University, Chengdu, China; ^2^Department of Urology, West China Hospital, Sichuan University, Chengdu, China

**Keywords:** home care, healthcare workers, need, latent class analysis, Chinese

## Abstract

**Background:**

The aging population has led to a surge in demand for home care, which has developed rapidly in China in recent years. However, there has been less empirical research into the needs of healthcare workers about providing home care. The purpose of this study was to explore the latent classes of healthcare workers' needs in primary health care institutions and to identify associated factors.

**Methods:**

From August 2021 to June 2022, a convenience sampling method was adopted to conduct a questionnaire survey on the workers of 62 primary healthcare institutions in Sichuan Province. Latent class analysis was used to categorize home care needs by Mplus 8.3. Multinomial logistic regression analysis was adopted to explore the influencing factors using SPSS 25.0.

**Results:**

A total of 1,152 healthcare workers were included in the study. Their needs for home care were classified into four latent classes: overall high need group (18.0%); overall low need group (34.8%); high training and low support need group (29.9%), and the high security and low training need group (17.3%). The factors influencing the different need categories included working area, professional title, role of medical workers, had participated in training about home care, and feelings about home care, with Class 1 as the reference group.

**Conclusion:**

Our findings indicate that primary healthcare workers have multifaceted needs for providing home care. Paying attention to their diverse needs can help optimize home care and enhance service capacity. Exploring the factors affecting needs can provide targeted support to healthcare workers to ensure the quality and continuity of home care services.

## 1 Introduction

Population aging is a major issue worldwide and a serious challenge for China. It is predicted that the number of people aged ≥60 years worldwide could reach nearly one in six in 2030 (1). China reported that ~18.70% of its population was aged 60 years or older in 2020 (2). Older people often face a general decline in physical function and an increased risk of chronic diseases such as diabetes, hypertension and cardiovascular disease. Meeting their growing demand for health care is a considerable challenge.

At present, care for older people includes formal care provided by medical institutions and informal care provided by family members. In recent decades, the “4-2-1” family structure (four grandparents, two parents and one child) emanating from the one-child policy and the phenomenon of young people congregating in big cities have led to a gradual weakening of family support, making it difficult to care for the older adult (3). Traditional informal care services cannot provide a high quality of life for the older adult. As a result, there is a growing need for formal care provided by health care teams. “Home care,” as a form of access to medical care, allows the older people to be cared for in a familiar place and is generally welcomed by the older adult (4, 5). The government also suggested that geriatric care should be provided by home care (80%), community care (15%), and institutional care (5%) (3). Home care has become an effective response to aging.

In 2021, the Sichuan Provincial Health Commission issued an announcement named “Sichuan Province on strengthening home medical services for the older adult work implementation plan (Trial)” (6). This plan explained home care, which referred to the provision of medical care, rehabilitation treatment, hospice care, Chinese medicine treatment and other home-based medical services to specific groups of people by medical personnel, with emphasis on older patients. The plan also encouraged primary health care institutions to vigorously develop home care and to continuously improve the content and form of the service. Home care is considered a vital supplement and effective auxiliary means of traditional hospital care. It can improve patient care and quality of life, reduce healthcare expenditures and the risk of cross-infection (7, 8). Some studies had shown that it can also reduce emergency room visits and unplanned readmissions (9, 10). For healthcare workers, home care can increase personal income, improve communication with patients and enhance emergency management skills.

Although some regions have achieved significant results, home care in China still faces many difficulties and challenges. As comorbidities and disability incapacities in old age are increasing year by year (11), the disease management and care of the older adult have become complex. Moreover, the uncertainty of the home environment creates greater challenges for medical personnel. This places higher demands on the inner quality and behavior of healthcare workers. However, some staff indicated that they were not adequately prepared to provide the service. They reported difficulties in safety precautions, emergency operations, emotional support, and dealing with challenging behaviors (12, 13). What can be done to help medical staff cope with the difficulties of service? First, identifying and knowing their specific needs is the first step to improving the problem. Understanding the needs of healthcare workers helps to identify the dilemmas they face and the support they need in the process of service. This will promote the rational use of limited health resources, build a home care service model that meets the characteristics of the national population, and promote the sustainable development of home care.

At present, home care services are mainly provided by the medical staff of primary healthcare institutions in China (6), such as general practitioner, nurses and rehabilitation therapists. The needs of these healthcare workers play a very important role in the provision and development of home care. Most of the existing studies had focused on the needs of patients (14, 15), with less attention given to the needs of healthcare workers. The little literature was mostly focused on the training needs of medical workers, and what other needs were not clear. Differences in hospital management models and medical resource allocation may lead to wide differences in the needs of medical personnel to provide home health care. If the needs of medical staff are assessed in the same way, or if only one need is focused on, problems with services are not well-addressed, leading to a waste of resources and inappropriate deployment of staff in the home care model.

In this study, we used latent class analysis (LCA) to clarify the different types of home care need among healthcare workers in primary care institutions and to identify associated factors. The results of this study will help to identify differences in the needs of healthcare workers and provide a basis for targeted support measures.

## 2 Methods

### 2.1 Data and sample

This was a multicenter survey conducted in Sichuan Province, China. Since the main institutions providing home care were primary health care institutions (16) and the national policies had encouraged primary institutions to expand home care service (17), this study took the lead in surveying the healthcare workers of primary health care institutions. These healthcare workers came from 62 primary health care institutions in seven cities, including Chengdu, Nanchong, Yibin, Panzhihua, Leshan, Guangyuan, and Suining. From August 2021 to June 2022, we used convenience sampling to include as many survey respondents as possible, with the following specific inclusion criteria: (1) obtained a professional qualification certificate; (2) a regular employee of the organization worked for more than 1 year; (3) known about home care or had already provided home care; and (4) voluntary participation.

Finally, a total of 1,152 healthcare workers participated in the survey, among which 33.9% were ≤ 30 years old, 41.1% were 31–40 years old, and the other 25.0% were>40 years old. The majority were females (91.1%). Approximately 39.0% worked for ≤ 5 years. The majority of them (71.6%) worked in urban areas. More than half of the respondents had a bachelor's degree as their highest level of education (55.0%) and a junior or lower title (59.8%). Nearly three-quarters (74.9%) had provided home care. More than half (52.4%) had no experience of working in a general hospital. Healthcare workers who participated in this survey were mainly nurses (55.0%), followed by doctors (28.3%). About 52.6% of the respondents had participated in home care training. Most people (76.9%) supported their workplaces to develop home care and over half (54.9%) felt better about home care (Table 1).

**Table 1:** Demographic characteristics of the participants (*n* = 1,152).

**Variable**	***n* (%)**
**Age (years)**	
≤ 30	390 (33.9)
31–40	474 (41.1)
>40	288 (25.0)
**Gender**	
Male	103 (8.9)
Female	1,049 (91.1)
**Working years**	
≤ 5 years	449 (39.0)
6–10 years	324 (28.1)
>10 years	379 (32.9)
**Working area**	
Urban areas	825 (71.6)
Rural areas	327 (28.4)
**The highest level of education**	
Secondary education	72 (6.3)
College degree	434 (37.7)
Bachelor's degree	635 (55.0)
Graduate degree	11 (1.0)
**Professional title**	
Junior title and below	689 (59.8)
Intermediate title	341 (29.6)
Senior title	122 (10.6)
**Had provided home care**	
Yes	863 (74.9)
No	289 (25.1)
**Working experience in general hospitals**	
Yes	548 (47.6)
No	604 (52.4)
**Role of medical workers**	
Doctor	326 (28.3)
Nurse	634 (55.0)
Other personnel	192 (16.7)
**Participated in training about home care**	
Yes	606 (52.6)
No	546 (47.4)
**Supporting workplaces to develop home care**	
Yes	886 (76.9)
No	266 (23.1)
**Feelings about home care**	
Not good	76 (6.6)
Average	444 (38.5)
Good	632 (54.9)

### 2.2 Measures

#### 2.2.1 Demographic characteristics

According to the needs of the topic setting and literature review, a general information questionnaire was developed on its own, which mainly included the age, gender, working years, working area, highest level of education, professional title, had provided home care, had working experience in general hospitals, role of medical workers, had participated in training about home care and supported their workplaces to develop home care. We also asked healthcare workers about their feelings about home care, and participants could respond on a scale of not good, average, and good.

#### 2.2.2 Questionnaire on home care need of healthcare worker

Based on Maslow's Hierarchy of Needs Theory (18) and previous researches related to the needs and experiences of medical professionals in providing home care (19–21), we designed a home care need questionnaire and developed a preliminary draft. Subsequently, we sought experts in home care related fields to form an expert panel to optimize the questionnaire's entries and evaluate content validity. The experts were contacted via email and WeChat communication software. A total of seven experts agreed to participate in this consultation. They were all at senior level, including three community clinical experts, three community nursing experts, and one nursing expert specializing in scale development. There were three PhDs, three master's degrees, and one bachelor's degree. They all had work experience for ≥15 years. All of them were engaged in home care services and had extensive experience in home-based services. Seven expert consultation questionnaires were completed and returned within the specified timeframe. The experts' motivation was 100%.

The final questionnaire consisted of 12 items in four main areas: training needs (three entries, theoretical knowledge, professional skills and communication skills training), safety needs (two entries, implementing risk prevention measures and safety monitoring of the unit and the platform), support needs (five entries, adequate provision of materials and equipment, legal safeguards, incentives in the unit, supportive cooperation from patients and their families and convenient transportation), and psychological needs (two entries, need to get respect and to embody self-worth). Each entry was answered with dichotomous answers (yes or no). The specific content of each item is shown in Table 2. For example, in terms of training needs, healthcare workers were asked if they had a need for conducting specialized theoretical training of home care. Respondents answered only by yes or no. The scale-level content validity index (S-CVI) was 0.97, and the item-level content validity index (I-CVI) ranged from 0.86 to 1.00 through the expert panel. Prior to the formal survey, 50 healthcare staff members were selected for the presurvey. All personnel reported that the questionnaire entries were clearly stated and the content was easy to understand and complete. Since the questionnaire entries were scored dichotomously, the Kuder-Richardson formula (Commonly KR-20) was used to calculate the reliability (22), which was measured as 0.813, indicating that the questionnaire had good reliability and validity.

**Table 2:** Home care needs of the participants (*n* = 1,152).

**Variable**	**Yes**	**No**
The need for conducting specialized theoretical training of home care.	500 (43.4%)	652 (56.6%)
The need for developing professional skills training of home care.	564 (49.0%)	588 (51.0%)
The need for improving communication skills of home care.	383 (33.2%)	769 (66.8%)
The need for implementing risk prevention measures, such as signing consent forms, purchasing insurance.	784 (68.1%)	368 (31.9%)
The need for security monitoring by units and platforms of home care.	538 (46.7%)	614 (53.3%)
The need for providing material and equipment support of home care.	137 (11.9%)	1,015 (88.1%)
The need for implementing legal protection of home care.	389 (33.8%)	763 (66.2%)
The need for promoting unit incentives of home care.	397 (34.5%)	755 (65.5%)
The need for obtaining supportive cooperation from patients and families.	478 (41.5%)	674 (58.5%)
The need for clarifying the transportation and routes of home care.	582 (50.5%)	570 (49.5%)
The need for getting respect.	390 (33.9%)	762 (66.1%)
The need for embodying self-worth.	291 (25.3%)	861 (74.7%)

### 2.3 Ethical approval

We first informed the heads of the institutions about the purpose and significance of this study and obtained their consent and cooperation. Then, the heads explained the instructions to the healthcare workers of their institutions and sent electronic questionnaires to be completed. The questionnaire was set to be completed only once for each IP address to avoid duplicate completion by the same personnel. Of course, this study was completed after obtaining informed consent from the participating organizations and personnel and signing the electronic informed consent form. The Biomedical Ethics Committee of West China Hospital of Sichuan University gave its approval to the study (Approval No. 2020-165).

### 2.4 Statistical analysis

LCA (Latent Class Analysis) is a method that uses dichotomous indicators to classify individuals with similar symptom profiles into categories (23). In this study, the 12 items of the Home Care Need Questionnaire were imported into Mplus 8.3 software for LCA. Starting with 1 model, the number of models was gradually increased until the optimal model was decided. The following parameters were considered: Akaike information criterion (AIC), Bayesian information criterion (BIC) and adjusted Bayesian information criterion (aBIC), which were widely used evaluation indexes. The smaller the value is, the better the model fit. The Lo-Mendell-Rubin likelihood ratio test (LMR) and Bootstrap likelihood ratio test (BLRT) were used to compare the differences in model fit. If the *P*-value was < 0.05, the model with k categories was better than the model with k-1 categories (24). The Entropy value was used to evaluate the quality of class classification. The closer to 1, the higher the classification accuracy. Subsequently, to explore the differences between the classes and factors influencing them, a one-way analysis of variance and a multinomial logistic regression analysis were performed using SPSS 25.0.

## 3 Results

### 3.1 Home care needs of healthcare workers

The needs for healthcare workers about home care are shown in Table 2. The highest need was for risk prevention measures, such as signing consent forms and purchasing insurance. The lowest need was for material and equipment support.

### 3.2 Latent class analysis and model fit

Mode fit statistics are shown in Table 3. The three indicators which included AIC, BIC, and aBIC decreased with the number of categories. The statistical values of LMR and BLRT supported all categories (*P* < 0.05). However, there was no significant increase in the value of the Entropy indicator after dividing into four or more categories, and when dividing into five and six categories, the probability of one of the categories was only 8%, a smaller percentage that may result in an inaccurate analysis of the sample. Based on the results obtained from the analysis, 4 categories were optimal.

**Table 3:** Fit indicators for different latent profile models.

**Model**	**AIC**	**BIC**	**aBIC**	**Entropy**	**LMR**	**BLRT**	**Class probability (%)**
Class1	17,432.842	17,493.433	17,455.318	-	-	-	1
Class2	16,028.835	16,155.066	16,075.658	0.830	0.000	0.000	305 (0.26)/847 (0.74)
Class3	15,542.366	15,734.237	15,613.537	0.791	0.000	0.000	529 (0.46)/246 (0.21)/377 (0.33)
Class4	15,326.686	15,584.198	15,422.206	0.764	0.005	0.000	207 (0.18)/401 (0.35)/345 (0.30)/199 (0.17)
Class5	15,240.222	15,563.374	15,360.089	0.766	0.007	0.000	445 (0.39)/151 (0.13)/251 (0.22)/209 (0.18)/96 (0.08)
Class6	15,179.336	15,179.336	15,323.552	0.747	0.000	0.000	271 (0.24)/271 (0.24)/180 (0.16)/128 (0.11)/98 (0.08)/204 (0.18)

AIC, Akaike information criterion; BIC, Bayesian information criterion; aBIC, adjusted Bayesian information criterion; LMR, Lo-Mendell-Rubin likelihood ratio test; BLRT, bootstrap likelihood ratio test.

### 3.3 Naming of latent classes

**Figure 1:**
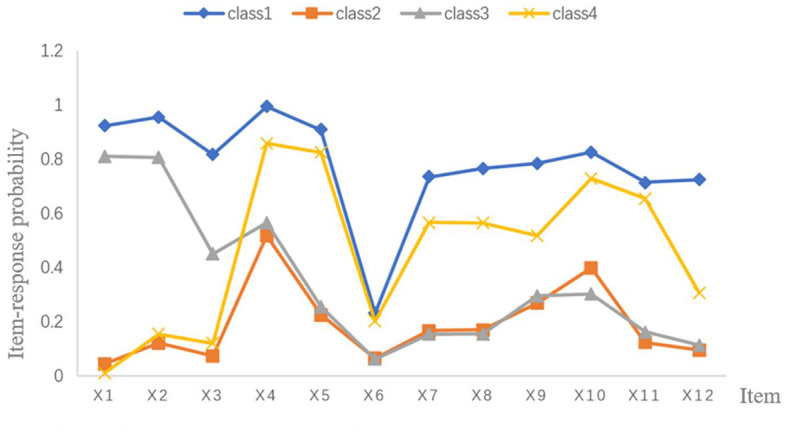
Item-response probabilities for four class model of home care need (1,152). Class 1 = The overall high need group (18.0%); Class 2 = The overall low need group (34.8%); Class 3 = The high training and low support need group (29.9%); Class 4 = The high security and low training need group (17.3%); X1 = The need for conducting specialized theoretical training of home care; X2 = The need for developing professional skills training of home care; X3 = The need for improving communication skills of home care; X4 = The need for implementing risk prevention measures, such as signing consent forms, purchasing insurance; X5 = The need for security monitoring by units and platforms of home care; X6 = The need for providing material and equipment support of home care; X7 = The need for implementing legal protection of home care; X8 = The need for promoting unit incentives of home care; X9 = The need for obtaining supportive cooperation from patients and families; X10 = The need for clarifying the transportation and routes of home care; X11 = The need for getting respect; X12 = The need for embodying self-worth.

The item-response probabilities in the classes are shown in Figure 1, and the probability of each item in four classes is shown in Table 4. Class 1 accounted for 18.0% (*n* = 207) of the sample, which had a greater probability in all 12 entries than the other categories. This category was defined as the overall high need group. Class 2 accounted for 34.8% (*n* = 401) of the sample. Most items in Class 2 had the lowest probability, while a few items had probabilities that were not the lowest, but not far from the minimum. Thus, we still named it as the overall low need group. Class 3 contained 29.9% (*n* = 345) of the sample. In this Class, the probability was higher at items 1 and 2, and lowest at items 6–8. This suggested that this category had a high need for home care training in theoretical and operational skills, but a low need for support such as material and equipment support, legal protection and the availability of incentives from the workplace. Therefore, this class was labeled as “high training and low support need group.” Class 4 contained 17.3% (*n* = 199) of the sample. We defined this class as the high security and low training need group. Because Class 4 had the lowest probability for the first three items about training (Theoretical training, operational skills training and communication skills training), and had the highest probability for items 4 and 5. The 4th and 5th items were about the need for security, including the implementation of risk prevention measures and security monitoring by platforms and units.

**Table 4:** Probability of each item in the four classes (*n* = 1,152).

**Item**	**Probability (%)**			
	**Class1**	**Class2**	**Class3**	**Class4**
The need for conducting specialized theoretical training of home care.	0.923	0.044	0.810	0.011
The need for developing professional skills training of home care.	0.955	0.121	0.806	0.154
The need for improving communication skills of home care.	0.816	0.073	0.449	0.120
The need for implementing risk prevention measures, such as signing consent forms, purchasing insurance.	0.994	0.518	0.566	0.858
The need for security monitoring by units and platforms of home care.	0.908	0.226	0.256	0.824
The need for providing material and equipment support of home care.	0.231	0.065	0.062	0.202
The need for implementing legal protection of home care.	0.734	0.167	0.154	0.567
The need for promoting unit incentives of home care.	0.765	0.171	0.155	0.564
The need for obtaining supportive cooperation from patients and families.	0.784	0.268	0.295	0.518
The need for clarifying the transportation and routes of home care.	0.825	0.399	0.301	0.728
The need for getting respect.	0.715	0.123	0.163	0.654
The need for embodying self-worth.	0.724	0.095	0.114	0.306

### 3.4 Demographic and related characteristics of each class

In the univariate analysis, the four latent classes were impacted by characteristics such as age, working years, working area, professional title, role of medical workers, had participated in training about home care, supported their workplaces to develop home care and feelings about home care. The results are displayed in Table 5.

**Table 5:** Demographic characteristics of the latent classes (*n* = 1,152).

**Category**	**Class1 (*n* = 157, 18.0%)**	**Class2 (*n* = 401, 34.8%)**	**Class3 (*n* = 345, 29.9%)**	**Class4 (*n* = 199, 17.3%)**	***X^2^***	***P***
**Age (years)**					18.522	0.005
≤ 30	68 (32.9%)	156 (38.9%)	122 (35.4%)	44 (22.1%)		
31–40	82 (39.6%)	155 (38.7%)	143 (41.4%)	94 (47.2%)		
>40	57 (27.5%)	90 (22.4%)	80 (23.2%)	61 (30.7%)		
**Gender**					2.347	0.504
Male	14 (6.8%)	34 (8.5%)	36 (10.4%)	19 (9.5%)		
Female	193 (93.2%)	367 (91.5%)	309 (89.6%)	180 (90.5%)		
**Working years**					14.822	0.022
≤ 5 years	70 (33.8%)	166 (41.4%)	144 (41.7%)	69 (34.7%)		
6–10 years	73 (35.3%)	116 (28.9%)	86 (24.9%)	49 (24.6%)		
>10 years	64 (30.9%)	119 (29.7%)	115 (33.3%)	81 (40.7%)		
**Working area**					7.905	0.048
Urban areas	141 (68.1%)	286 (71.3%)	240 (69.6%)	158 (79.4%)		
Rural areas	66 (31.9%)	115 (28.7%)	105 (30.4%)	41 (20.6%)		
**The highest level of education**					6.892	0.648
Secondary education	7 (3.4%)	28 (7.0%)	25 (7.2%)	12 (6.0%)		
College degree	75 (36.2%)	155 (38.7%)	133 (38.6%)	71 (35.7%)		
Bachelor's degree	124 (59.9%)	214 (53.4%)	183 (53.0%)	114 (57.3%)		
Graduate degree	1 (0.5%)	4 (1.0%)	4 (1.2%)	2 (1.0%)		
**Professional title**					19.764	0.003
Junior title and below	115 (55.6%)	261 (65.1%)	215 (62.3%)	98 (49.2%)		
Intermediate title	71 (34.3%)	96 (23.9%)	101 (29.3%)	73 (36.7%)		
Senior title	21 (10.1%)	44 (11.0%)	29 (8.4%)	28 (14.1%)		
**Had provided home care**					2.528	0.470
Yes	150 (72.5%)	301 (75.1%)	255 (73.9%)	157 (78.9%)		
No	57 (27.5%)	100 (24.9%)	90 (26.1%)	42 (21.1%)		
**Working experience in general hospitals**					1.985	0.576
Yes	104 (50.2%)	182 (45.4%)	162 (47.0%)	100 (50.3%)		
No	103 (19.8%)	219 (54.6%)	183 (53.0%)	99 (49.7%)		
**Role of medical workers**					18.138	0.006
Doctor	38 (18.4%)	113 (28.2%)	107 (31.0%)	68 (34.2%)		
Nurse	130 (62.8%)	223 (55.6%)	174 (50.4%)	107 (53.8%)		
Other personnel	39 (18.8%)	65 (16.2%)	64 (18.6%)	24 (12.1%)		
**Participated in training about home care**					18.949	< 0.001
Yes	85 (41.1%)	234 (58.4%)	191 (55.4%)	96 (48.2%)		
No	122 (58.9%)	167 (41.6%)	154 (44.6%)	103 (51.8%)		
**Supporting workplaces to develop home care**					63.173	< 0.001
Yes	144 (69.6%)	339 (84.5%)	286 (82.9%)	117 (58.8%)		
No	63 (30.4%)	62 (15.5%)	59 (17.1%)	82 (41.2%)		
**Feelings about home care**					113.488	< 0.001
Not good	20 (9.7%)	16 (4.0%)	8 (2.3%)	32 (16.1%)		
Average	108 (52.2%)	126 (31.4%)	109 (31.6%)	101 (50.8%)		
Good	79 (38.2%)	259 (64.6%)	228 (66.1%)	66 (33.2%)		

A multinomial logistic regression analysis was carried out using the four classes as the dependent variable and the statistically significant in one-way analysis of variance as independent variables (*P* < 0.05), with Class 1 as the reference group to analyze the variables connected to home care need. The outcomes are shown in Table 6. According to the results, the overall need for healthcare staff at the intermediate level was lower and more likely to be in Class 2 compared to the junior and lower titles. Doctors and healthcare workers who had participated in training were more likely to be in Class 1 than professionals such as rehabilitators, pharmacists, and nurses. In addition, feelings about home health care services varied, as did needs. Those who held a good feeling had higher needs, especially compared to Class 2 and Class 3. Finally, those working in rural areas had a higher need for security and were more likely to be in Class 4.

**Table 6:** Multinomial logistic regression analysis (*n* = 1,152).

**Variables**	**Class2 vs. Class1**		**Class3 vs. Class1**		**Class4 vs. Class1**	
	***OR***	***95% CI***	***OR***	***95% CI***	***OR***	***95% CI***
**Age (years)**						
≤ 30	Ref					
31–40	0.957	0.591–1.549	0.867	0.526–1.430	0.574	0.323–1.021
>40	1.520	0.798–2.897	1.386	0.717–2.680	0.853	0.407–1.785
**Working years**						
≤ 5 years	Ref					
6–10 years	1.158	0.747–1.795	1.402	0.887–2.216	1.623	0.960–2.746
>10 years	0.807	0.474–1.376	0.737	0.428–1.267	0.919	0.512–1.649
**Working area**						
Rural areas	Ref					
Urban areas	0.764	0.520–1.122	0.845	0.570–1.254	0.575^*^	0.360–0.920
**Professional title**						
Junior title and below	Ref					
Intermediate title	1.700^*^	1.071–2.699	1.489	0.932–2.380	1.332	0.799–2.220
Senior title	1.211	0.588–2.497	1.894	0.885–4.051	1.016	0.462–2.236
**Role of medical workers**						
Other personnel	Ref					
Doctor	0.441^**^	0.251–0.775	0.462^**^	0.262–0.815	0.368^**^	0.190–0.713
Nurse	1.032	0.836–1.669	1.343	0.824–2.188	0.780	0.433–1.405
**Participated in training about home care**						
No	Ref					
Yes	0.689^*^	0.478–0.994	0.780	0.536–1.137	0.639^*^	0.418–0.978
**Supporting workplaces to develop home care**						
No	Ref					
Yes	0.766	0.476–1.233	0.967	0.596–1.570	1.524	0.930–2.498
**Feelings about home care**						
Not good	Ref					
Average	0.823	0.388–1.745	0.397^*^	0.161–0.978	1.571	0.801–3.080
Good	0.348^*^	0.155–0.784	0.148^***^	0.057–0.384	1.574	0.720–3.442

OR, Odds ratio; 95% CI, 95% Confidence Interval; Ref, Refer.

^*^p < 0.05.

^**^p < 0.01.

^***^p < 0.001.

## 4 Discussion

This study classified the needs of home health care services of medical staff through the LCA approach. Four different types of home care needs were identified according to four dimensions: training needs, safety needs, support needs and psychological needs. Our research showed that the different types of medical staff needs were related to their working area, professional title, role, whether they participated in home care training and the feeling about home care. As the main provider of home health care, medical staff should be concerned about their job-related needs, which are closely related to their willingness and quality of service delivery. The results of this study can also provide reference for subsequent targeted intervention.

### 4.1 General information and home care needs of healthcare workers

A total of 1,152 healthcare workers were surveyed, with one-third aged ≤ 30 years and had ≤ 5 years of working experience (39.0%), More than half (55.0%) had a bachelor's degree and a junior or lower title (59.8%). This indicated a younger population in this survey. The majority (91.1%) were female, which may be due to the fact that most healthcare workers in this survey were nurses (55.0%), followed by doctors (28.3%). Healthcare workers were relatively more female in China. And the salary packages in primary institutions were less compared to those in large general hospitals. Men tend to be the main bearers of family burdens, so they usually choose large hospitals with better salary. About three quarters had already provided home care (74.9%) and worked in urban area (71.6%). This was also related to the fact that the current location of home care was still predominantly urban, where the need and willingness to pay for home care were higher. More than half of them had attended training related to home care (52.6%) and had a better feeling about home care (54.9%), though not having working experience in general hospitals (52.4%). Most of them (76.9%) were also supporting their workplace to develop home care. This suggested that they had a positive attitude toward the development of home care.

As can be seen from the needs of healthcare workers, the highest need was for risk prevention measures, while the need for material and equipment support is minimal. This indicated that most institutions were currently able to provide enough material and equipment support for home care, but there were deficiencies in security management, which was consistent with the findings of other scholars' surveys of home care (25, 26). The existing safety management was some simple measures such as signing an agreement with the patient and keeping the phone number of family members. There is a serious lack of comprehensive measures that could allow for constant monitoring and immediate activation of safety precautions. Institutions should make full use of electronic devices to monitor staff movements on the platform. Insurance and one-button alarm devices should also be made for the staff, so that the nearest support and rescue team can be activated immediately in the event of an emergency.

### 4.2 The latent classes of home care needs

By analyzing the 12 entries of the questionnaire, the needs of healthcare workers were divided into four classes: overall high need group (Class 1), overall low need group (Class 2), high training and low support need group (Class 3), and high security and low training need group (Class 4). The proportion of Class 2 (34.8%) was the largest, and the overall need for home care in this group was low in all cases. Considering the late start of home care in China, the awareness of healthcare workers may not be high. The majority of healthcare professionals in this survey had already provided home care, and their needs may be relatively low due to a greater familiarity with the process and content of the service. Class 4 (17.3%) had the smallest percentage, and this group had a high need for security and a relatively small need for training. A higher proportion of healthcare workers in Class 4 were older, had longer working years, and had middle to senior title. There was also a preponderance of general hospital employment relative to other groups in Class 4. These people had a certain theoretical and skill base, which could explain their lower need for training. Compared with other classes, healthcare workers who did not support their institutions in carrying out services and had bad feelings about home care accounted for a larger proportion in Class 4, which was related to higher security need. Previous studies had also shown that security was one of the current issues that needed to be focused on and addressed (25, 26), which was more consistent with the high security need of this study. Institutions should take safety precautions to avoid accidents, including filing patient information, wearing safety devices, and making a companion visit.

### 4.3 Association between socio-demographic and latent class membership

As homecare workers exist in different institution and environment, their needs for providing home care are inevitably influenced by multiple factors. The results of the univariate analysis showed that the different classes of need were mainly influenced by eight factors. In the multifactor analysis, working area, professional title, role of healthcare workers, participated in training about home care and feelings about home care had a significant effect on the different classes of need.

Healthcare workers with junior titles and below were in the overall high need group (Class 1), while those with an intermediate professional title had a statistically significant effect on the likelihood of being in the overall low need group (Class 2). The staff of primary health care organizations in Sichuan Province was still dominated by low educational attainment and low professional titles (27). At the same time, the limited size and staffing of the organization made the healthcare workers with higher titles more engaged in management and teaching related work. Workers with an intermediate professional title had fewer opportunities to provide services directly, which may be one of the reasons for the lower need. Healthcare workers at the junior level and below had weaker basic knowledge and more opportunities to provide services. Their need for services was higher, contrary to the results of a previous survey on the home care need provided by nurses in China (28). The reason for this may be that the target population of the two studies was quite different, with the present study being dominated by healthcare workers in primary healthcare institutions, which including rehabilitators and pharmacists, whereas the previous study surveyed nurses in various large hospitals. The healthcare workers at junior level and below, who were currently the main providers of home care, were crucial to ensuring patient safety and improving the quality of services. Institutions should conduct surveys about their need and pay attention to their suggestions and unmet needs of junior level staff. The competent department in the region regularly investigate the status of implementation. Policies can also be adopted to bring in talents with high titles to enhance the overall professionalism of the primary health care institutions.

Among the different types of medical personnel, doctors were less likely to be in Class 2 or Class 3 or Class 4, and more likely to be in Class 1 (overall high need group). As seen from the distribution of human resources in primary healthcare institutions in Sichuan Province, doctors generally had higher academic qualifications and were more willing to continuously pursue self-improvement, compared with nurses, rehabilitators, psychological counselors and other personnel (27). In particular, since 2016, the state had vigorously promoted family doctor contract services (FDCS), which was a doctor-centered team carring out whole-course management of residents' health to prevent and control diseases (29). As the important role of home care, doctors not only have to provide services at home, but also communicate with other staff members to understand the patient's treatment and manage the patient's health throughout the entire process (30, 31), which will result in a greater need for training, safety and support in the provision of services. Doctors in primary care institutions are usually general practitioners who need to know all disease-related knowledge and operations. When providing home care, they are also usually faced with risky operations such as puncture and prescribing medicines. The high need demonstrated by doctors also reflected the high pressures and high standards they faced. Institutions should address the needs of different personnel based on individualized support measures. In addition to guaranteeing their basic security needs, positive incentives should be used to enhance the self-fulfillment and psychological support of medical personnel.

Compared with trained medical staff, those who did not have home care training were more likely to be in Class 2 (overall low need group) and Class 4 (high security and low training need group). Those who had attended training were more likely to fall into Class 1 (overall high need group). Initially, home care was developed gradually by rural doctors visiting patients to provide treatment, and there was no mandatory requirement to attend training (32). In recent years, home care has been gradually standardized. Corresponding requirements had been imposed on service providers. Regular participation in relevant training to enhance practical behavior had become the focus of some units' assessment (6). Those who had not participated in training were not highly motivated about home care. In our study, most healthcare workers who were not trained had already provided home care. They had a certain service base, and they focused on safety rather than training. With the increasing aging population, home care is a major trend. The adoption of relevant training is the beginning of medical personnel's understanding of home health care. We can learn from the experience of foreign countries. For example, Japan had incorporated home care into the school curriculum, which could enhance the understanding of home care and fully motivate medical personnel to provide quality services.

Healthcare professionals who felt good about home care had higher overall needs (Class 1), while those who felt average and worse were more likely to be in Class 2 and Class 3. This suggested that staff feelings were closely related to the needs. It may be that those who had a good feeling about home care were more motivated and inclined to enhance the quality of the service and improve the patient experience through training and the adoption of safety measures. Those who did not feel well may be psychologically repulsed by the provision of home care and may be less enthusiastic about the associated need for the service. In this study, ~54.9% of the healthcare workers explicitly indicated that they felt good, and <½ of them felt bad or fair about home care, which was in line with the results of a Chinese study on mixed feelings about nurse home visits (33). The feeling of home care can be affected by a variety of factors which may be related to the imperfection of regulations, incomplete safeguards, higher safety risks and poor patient compliance (34, 35). The authorities should focus on the reasons for their bad feelings and propose targeted improvement measures, which can also enhance the enthusiasm and motivation of medical staff to provide home care. Institutions should make full use of the mature experience in the development of home care. Relevant laws and regulations should be improved to protect the legitimate rights of medical personnel. In the process of providing services, social support can be strengthened to improve patient compliance, so as to promote the smooth progress of home medical care (36, 37). And their needs should also be encouraged to express freely, which will also promote the sustainability of home care.

The staff working in rural areas were more likely to be in Class 4 (high security and low training need). while those working in urban areas were more likely to be in Class 1. This suggested that the need for safety was more prominent among health workers working in rural areas, while the need for training was relatively small. Rapid economic and social development led to a shift of the young population to cities. The rural population was gradually decreasing (38). People's need for medical treatment and health maintenance is growing. Many patients, especially those suffering from major diseases, usually choose large hospitals in cities for treatment and rehabilitation. Medical personnel in rural areas mainly perform simple treatments and operations. The widespread use of the internet has also made training easier, and the need for training by healthcare workers is relatively less urgent (39). This was in contrast to the results of Zhou's systematic evaluation (32), which may have contributed to the different results because the systematic evaluation included 10 studies before 2010, when training was not yet widely available. In addition, due to the poor economic development of rural areas, the inconvenience of road transportation, and the limited material and equipment of the institutions, healthcare workers attach great importance to the need for safety. A study showed that only 17.60% of healthcare workers in primary health care organizations assessed the risk of home visits, and < 30% provided safety support measures, which made safety become an issue of great concern (35). The unequal distribution of resources between urban and rural areas created an urgent need for the state to provide policy support for rural areas. This included encouraging general hospitals and social capital to move into rural areas, strengthening infrastructure, providing adequate supplies and safety equipment, and enhancing the accessibility of rural care services through networked information technology. Home care safety is a major problem in multiple countries. The configuration of security facilities in rural areas is far less than that in cities, and its security problems are more worrying. Existing security measures in most institutions are unable to meet the higher needs of medical staff. Advanced equipment has also not achieved regional coverage yet. How to improve the safety configuration of rural areas and reduce the safety concerns of rural medical personnel remains to be further explored and to work together for scholars.

Home care services are being piloted in several cities in China, but a mature service system has yet to be formed. Both the government and organizations are making continuous attempts and efforts to strike a balance in the development of home care. However, how to protect the rights of the provider, the provided and the organizations, and how to ensure that the needs of the provider are adequately taken care of and reasonably met, requires the joint appeal of the medical profession.

### 4.4 Limitations

This study first reported on the latent classes of home care need for healthcare workers and its predictors in China. However, there were some limitations. First, although this study endeavored to broaden the scope of the survey by selecting different cities, they were all still located in Sichuan Province, and geographical and population limitations may affect further generalization of the results. The participants in this survey were predominantly women and working in urban areas. More men and healthcare workers working in rural areas should be included in subsequent studies to promote a relatively balanced sample. Second, this was only a cross-sectional survey, which did not allow for further causality. As needs may change over time, a longitudinal study could be conducted to explore the trajectory of changes in needs for precise interventions.

## 5 Conclusion

Four distinct latent classes of healthcare workers of primary healthcare institutions were identified with regard to home care need. Our findings provided useful implications for managers to better understand the classes of home care need and help them provide timely supportive intervention. Different classes of needs had their own characteristics and influencing factors. Institutions should take an active interest and target support according to the corresponding needs. Besides, the contents of this study may provide ideas for formulating related courses and training.

## Data Availability

The raw data supporting the conclusions of this article will be made available by the authors, without undue reservation.

## References

[1] World Health Organization. Ageing and Health. (2022). Available at: https://www.who.int/news-room/fact-sheets/detail/ageing-and-health (accessed December 2, 2023).

[2] National Bureau of Statistics. China Population Census Yearbook. China Statistics Press (2021). Available at: https://www.stats.gov.cn/sj/pcsj/rkpc/7rp/zk/indexce.htm

[3] Fang EF Scheibye-Knudsen M Jahn HJ Li J Ling L Guo H. A research agenda for aging in china in the 21st century. Ageing Res Rev. (2015) 24:197–205. 10.1016/j.arr.2015.08.003 26304837 PMC5179143

[4] Drennan VM. More care out of hospital? A qualitative exploration of the factors influencing the development of the district nursing workforce in England. J Health Serv Res Policy. (2019) 24:11–8. 10.1177/1355819618769082 29754532 PMC6304681

[5] Zhou J Walker A. The impact of community care services on the preference for ageing in place in urban china. Health Soc Care Community. (2021) 29:1041–50. 10.1111/hsc.13138 32783285

[6] Sichuan Provincial Health Commission. Sichuan Province on Strengthening Home Medical Services for the Elderly Work Implementation Plan. (2021). Available at: http://wsjkw.sc.gov.cn/scwsjkw/sclljk/2021/5/28/e087ec5b275d42acaab752c9c1daf24a.shtml

[7] Gomez-Centurion I Oarbeascoa G Garcia MC López Fresneña MC Martínez Carreño MJ Escudero Vilaplana V. Implementation of a hospital-at-home (HAH) unit for hematological patients during the COVID-19 pandemic: safety and feasibility. Int J Hematol. (2022) 115:61–8. 10.1007/s12185-021-03219-2 34553338 PMC8457036

[8] Schuchman M Fain M Cornwell T. The resurgence of home-based primary care models in the United States. Geriatrics. (2018) 3:41. 10.3390/geriatrics3030041 31011079 PMC6319221

[9] Lai FTT Wong EL Tam ZP Cheung AW Lau MC Wu CM. Association of volunteer-administered home care with reduced emergency room visits and hospitalization among older adults with chronic conditions: a propensity-score-matched cohort study. Int J Nurs Stud. (2022) 127:104158. 10.1016/j.ijnurstu.2021.104158 35092873

[10] Andriastuti M Halim PG Mulyati T Bangun M Widodo DP. Palliative home visit intervention and emergency admission in pediatric cancer children: a randomized controlled trial. Curr Pediatr Rev. (2024) 20:194–9. 10.2174/1573396318666220928112910 36173046

[11] Hajat C Kishore SP. The case for a global focus on multiple chronic conditions. Br Med J Glob Health. (2018) 3:874. 10.1136/bmjgh-2018-000874 29989034 PMC6035500

[12] SoonOk K Hee BS. Care workers' experiences of emergencies with Korean older adults: a qualitative descriptive study. J Korean Gerontol Nurs. (2022) 24:151–61. 10.17079/jkgn.2022.24.2.15119940089

[13] Fernandez-Medina IM Ruiz-Fernandez MD Galvez-Ramirez F Martínez-Mengíbar E Ruíz-García ME Jiménez-Lasserrotte MDM. The experiences of home care nurses in regard to the care of vulnerable populations: a qualitative study. Healthcare. (2022) 10:21. 10.3390/healthcare10010021 35052185 PMC8774707

[14] Wang Z Liu Z. Latent classes and related predictors of demand for home-and community-based integrated care for older Chinese adults. Front Public Health. (2023) 11:9981. 10.3389/fpubh.2023.1109981 37427265 PMC10326318

[15] Kim H-J Lim J-Y Jang S-N. Korean primary health care program for people with disabilities: do they really want home-based primary care? BMC Health Serv Res. (2023) 23:1086. 10.1186/s12913-023-10102-9 37821901 PMC10568830

[16] Zhou LL Liu SZ Li H. Investigation on the situation of primary health care institutions and home-based medical service in Sichuan Province. Chin Gen Prac. (2024) 27:433–9. 10.12114/j.issn.1007-9572.2023.0018

[17] National Health Commission. Notice on strengthening the work of home medical services for the elderly (2020). Available at: http://www.nhc.gov.cn/yzygj/s7653pd/202012/19a2617ba8e641bea9ac2472ea04c82a.shtml.

[18] Maslow AH. A theory of human motivation. Psychol Rev. (1943) 1943:h0054346. 10.1037/h0054346

[19] Pang YJ Tang XP Hong B Zhou LS. Field research on service recipients, job content and work experience of home health nurse working in community health centers in Shanghai. J Nurs Sci. (2018) 33:66–9. 10.3870/j.issn.1001-4152.2018.12.066

[20] Zhong Y Chen SH Lv XY Shen Y Lv XX. Qualitative research on the willingness and needs of community nurses in the city to participate in the “internet plus wound care service”. J Nurs Train. (2021) 36:193–6. 10.16821/j.cnki.hsjx.2021.03.001

[21] Hassankhani H Rahmani A Best A Taleghani F Sanaat Z Dehghannezhad J. Barriers to home-based palliative care in people with cancer: a qualitative study of the perspective of caregivers. Nurs Open. (2020) 7:1260–8. 10.1002/nop2.503 32587746 PMC7308678

[22] Tan S. Misuses of KR-20 and Cronbach's alpha reliability coefficients. Egit Bilim. (2009) 34:101–12.

[23] Li J Sun Y Maccallum F Chow AYM. Depression, anxiety and post-traumatic growth among bereaved adults: a latent class analysis. Front Psychol. (2021) 11:575311. 10.3389/fpsyg.2020.575311 33519589 PMC7844091

[24] Feng Y Harring JR. Structural equation modeling: applications using Mplus. Psychometrika. (2020) 85:526–30. 10.1007/s11336-020-09706-5

[25] Lindberg C Fock J Nilsen P Schildmeijer K. Registered nurses' efforts to ensure safety for home-dwelling older patients. Scand J Caring Sci. (2023) 37:571–81. 10.1111/scs.13142 36582025

[26] Yoshimatsu K Nakatani H. Attitudes of home-visiting nurses toward risk management of patient safety incidents in japan. BMC Nurs. (2022) 21:139. 10.1186/s12912-022-00905-2 35668490 PMC9169385

[27] Zhou ZF Yang SJ Gao B Wu NW Ma H Li X. Current situation of health human resource allocation in Sichuan Province from the regional perspective. J Prev Med Inf. (2022) 38:538–43.

[28] Ma G Hou J Peng S Luo L Xu R Liu Y. Nurses' willingness and demand for internet plus home care services and its influencing factors in different levels of hospitals in china - a nationwide s urvey. Risk Manag Healthc Policy. (2022) 15:1395–405. 10.2147/RMHP.S367412 35911086 PMC9326896

[29] Li J Li J Fu P Chen Y Tang X Li Z. Willingness of patients with chronic disease in rural china to contract with family doctors: implication for targeting characteristics. Bmc Fam Pract. (2021) 22:203. 10.1186/s12875-021-01553-2 34649515 PMC8518214

[30] Chang-O K Hong J Cho M Choi E. Developing a model of home-based primary care in South Korea: a 1.5-year case study. J Korean Gerontol Soc. (2020) 40:1403–28. 10.31888/JKGS.2020.40.6.1403

[31] Zhang Z Zhang R Peng Y Zhai S Zhang J Jin Q. Barriers and facilitators of family doctor contract services in caring for disabled older adults in Beijing, China: a mixed methods study. Br Med J Open. (2023) 13:70130. 10.1136/bmjopen-2022-070130 37263682 PMC10255286

[32] Zhou L Wei X Wu Y Deng X Xu M Shang X. Preferences for training needs of village doctors in China: a systematic review. Fam Pract. (2023) 2023:cmad063. 10.1093/fampra/cmad063 37300310

[33] Wang LT Tang LJ Yue LC Li YH Xu XH Zhao YQ. The study on work experience of nurses in internet home-care service. Chin J Nurs. (2020) 55:1067–71. 10.3761/j.issn.0254-1769.2020.07.02038809583

[34] Morioka N Kashiwagi M. Adverse events in home-care nursing agencies and related factors: a nationwide survey in Japan. Int J Environ Res Public Health. (2021) 18:52546. 10.3390/ijerph18052546 33806436 PMC7967548

[35] Gao H Liu SZ Li H. The safety support and willingness of providing home care services by medical staff of primary health care institutions. Chin Gen Prac. (2022) 25:4326–31. 10.12114/j.issn.1007-9572.2022.0513

[36] Yang Z Sun L Sun Y Dong Y Wang A. A conceptual model of home-based cardiac rehabilitation exercise adherence in patients with chronic heart failure: a constructivist grounded theory study. Patient Prefer Adher. (2023) 17:851–60. 10.2147/PPA.S404287 36999162 PMC10044075

[37] Yang Z Jia H Zhang F Huang H Hao X Wang A. A behavioural driving model of adherence to home-based cardiac rehabilitation exercise among patients with chronic heart failure: a mixed-methods study. J Clin Nurs. (2024) 33:531–42. 10.1111/jocn.16901 37881110

[38] Rodriguez-Soler R Uribe-Toril J Valenciano JD. Worldwide trends in the scientific production on rural depopulation, a bibliometric analysis using bibliometrix r-tool. Land Use Policy. (2020) 97:104787. 10.1016/j.landusepol.2020.104787

[39] Xin H Zhang H Wang D Zhang B Cao X Feng B. The effect of WeChat-based training on improving the knowledge of tuberculosis management of rural doctors. J Clin Tuberc Other Mycobact Dis. (2021) 25:100266. 10.1016/j.jctube.2021.100266 34458591 PMC8379341

